# Combinatorial Approaches With Checkpoint Inhibitors to Enhance Anti-tumor Immunity

**DOI:** 10.3389/fimmu.2019.00999

**Published:** 2019-05-22

**Authors:** Barbara Seliger

**Affiliations:** Institute of Medical Immunology, Martin Luther University Halle-Wittenberg, Halle (Saale), Germany

**Keywords:** immune checkpoints, immunotherapy, tumor, inhibitors, biomarkers

## Abstract

Treatment of cancer patients has been recently revolutionized by the application of various immunotherapeutics. However, the response rates are still limited ranging between approximately 20 and 40% suggesting that combinations of immunotherapy with conventional treatment, like chemotherapy, radiation, epigenetic modulators, targeted therapies using small molecules as well as other (immuno) therapeutics, might be an option to increase systemic anti-tumor immunity. It is postulated that different non-immune based therapies in combination with immunotherapies could reprogram the immune suppressive tumor microenvironment and enhance the immunogenicity of tumor cells leading to an improved therapeutic efficacy and a better patients' outcome. Despite there exist various examples of increased objective responses achieved by adding these different therapies to immunotherapies, strategies for rational and evidence-based design of checkpoint inhibitor combinations to maximize the clinical benefit for patients are urgently required. Therefore, the main purpose of this review is to summarize recent results obtained from experimental models and clinical trials to enhance tumor immunogenicity by combining immunotherapy with other therapeutic options to maximize patients' outcome and minimize adverse events.

## Introduction

During the last decade novel tumor immunotherapeutic approaches have recently revolutionized the cancer treatment. In particular the clinical success of monoclonal antibodies (mAb) directed against immune checkpoint (iCP) molecules, such as the T lymphocyte antigen 4 (CTLA-4) and the programmed cell death protein 1/programmed cell death ligand 1 (PD-1/PD-L1) pathway, was a breakthrough achievement resulting in the Nobel Price of Medicine 2018. The anti-CTLA-4 mAb Ipilimumab was the Food and Drug Administration (FDA) approved checkpoint inhibitor (iCPI) followed by approval of Pembrolizumab, Nivolumab, and Cemiplimab directed against PD-1, in 2014, 2016, or 2018, respectively, for non-small cell lung cancer (NSCLC), melanoma, renal cell carcinoma (RCC), bladder cancer and/or squamous cell skin cancer and the anti-PD-L1 mAbs Durvalumab, Atezolizumab, and Avelumab in 2017 after promising results in NSCLC, urothelial carcinoma and Merkel cell carcinoma (1–3). Currently, a number of other “next generation” iCPIs directed against e.g., the lymphocyte-activation gene 3 (LAG-3), the T cell immunoglobulin and mucin domain-3 (TIM-3) and B7-H4, are tested in experimental models and/or clinical trials (4, 5). Despite the rapid progress in approvals for iCPIs in an expanding spectrum of malignancies, there exists accumulating evidence that approximately only one-third of patients achieve a durable long-term response as stand-alone intervention with iCPIs (6, 7). This might be due to primary and acquired resistance and thus limit the efficacy of the treatment (8–10) suggesting that a high frequency of patients do not respond to iCPI alone. In contrast, recent efforts combining iCPIs with conventional and other (immuno)therapies achieved response rates of over 50% (11, 12), since they not only mediate anti-neoplastic effects by cytotoxic and cytostatic mechanisms, but also by local as well as systematic modulation of immunological functions (13, 14).

Currently, a number of preclinical and clinical trials for iCPIs across all tumor types coupled with a second immunotherapeutic modality or combined with e.g., chemotherapy, targeted therapies, radiation therapy (RT), epigenetic modulators, inhibitors of histone deacetylases (HDAC), DNA methyltransferases (DNMT), or cyclin-dependent kinases 4 and 6 (CDK4/6), are conducted (13, 15).

**Figure 1:**
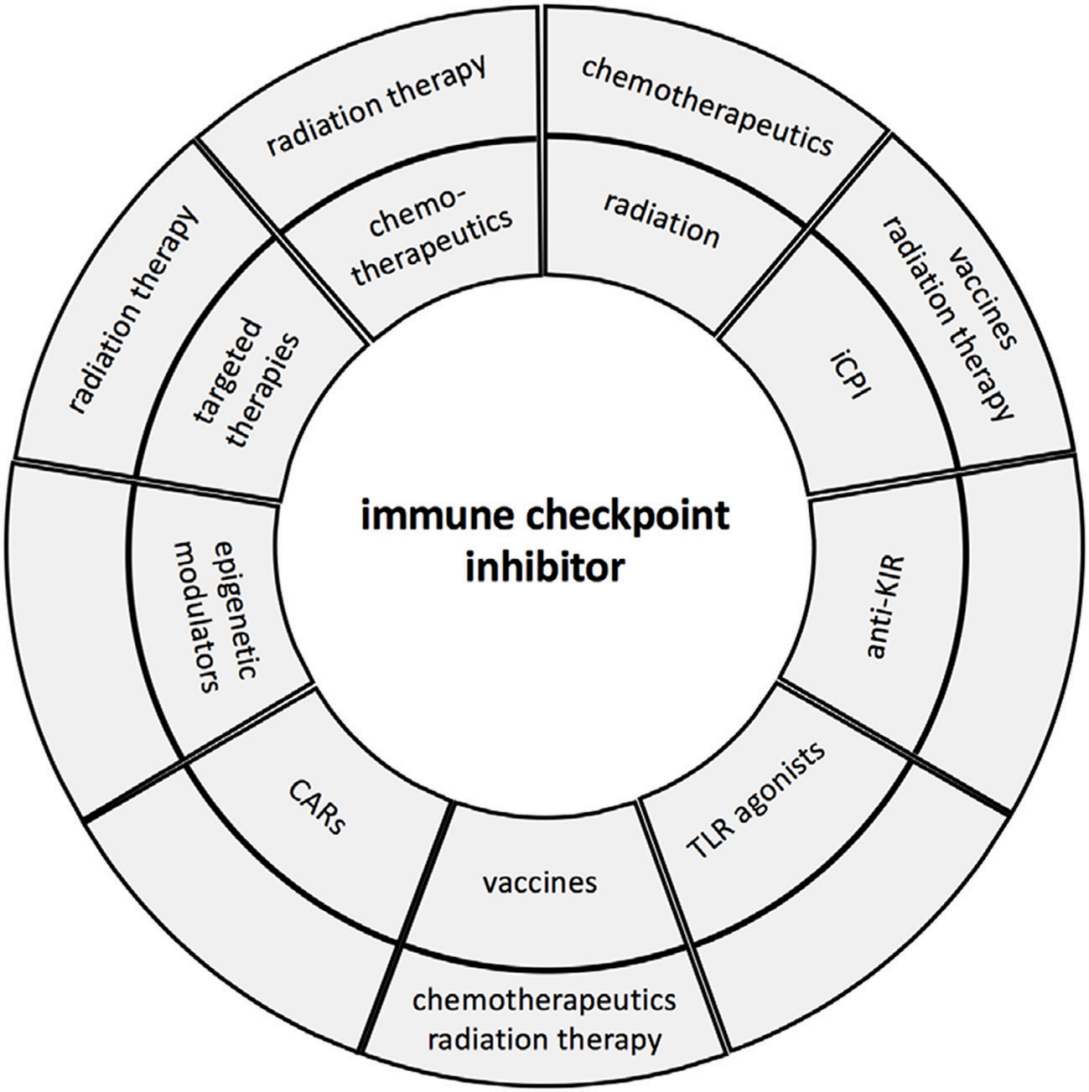
Immune check point inhibitor therapy and combinations. The major combinations of iCPIs and other therapies are summarized and presented in the first circle, while the second circle illustrates the triple combinations currently in clinical trials.

So far, 7 iCPIs and 1 combination immunotherapy regimen have been approved by the FDA since 2011 (16, 17). However, it is very obvious that these approaches will increase by rationally designed synergistic combinations based on an individualized patients' setting (14). Therefore, biomarkers are urgently required (i) to select patients who will benefit and respond to treatment and (ii) to identify the best combinations of agents for each patient to improve response rates, enhance treatment efficacies and mitigate toxicities (18, 19). The aim of these combinations are to enhance effector function of immune cells leading to tumor elimination, to modulate the immune suppressive tumor microenvironment (TME), to recruit T cells to the tumor, and to revert immune escape mechanisms (20). It is suggested that overcoming the different resistance mechanisms might be one major key for enhancing the efficacy of immunotherapies. This report summarizes the rational for the design of some promising regimens combining iCPIs with other treatment options and future strategies in order to attack the primary tumor, but also cancer cells within the patients' body including cancer stem cells, metastasis and circulating tumor cells (Figure 1).

## Combination of Immunotherapies With Other Immunotherapies

Due to the increase in available iCPIs and other immunotherapeutics, such as novel iCPIs, e.g., antagonistic antibodies directed against LAG-3, V-domain Ig suppressor of T cells activation (VISTA), and T cell immunoglobulin and mucin domain-3 (TIM-3) or activating antibodies against e.g., OX40, 4-1BB, ICOS, and CD40, novel vaccines, cytokines, anti-killer inhibitory receptors (KIR), oncolytic viruses, and cellular therapies, an abundance of possibilities exists, which are currently studied in animal models and/or clinical trials (21, 22). The rational for these combinations are the distinct molecules, but complementary pathways, which are targeted and therefore might result in synergistic effects. The following part briefly summarizes some selected major concepts.

### Combinations of iCPIs With Other iCPIs

Inhibition of CTLA-4 and PD-1 act on distinct pathways, e.g., central vs. peripheral immunity, the blockade of both pathways is complementary suggesting that simultaneous inhibition of these targets have additional anti-tumor activity (23). The first iCPI combination employed was Ipilimumab with anti-PD-1 in melanoma resulting in an increased efficacy, but also high levels of adverse events (23, 24). Examples of other iCPI combination strategies include anti-PD-1 and anti-PD-L1 antibodies in combination with established or novel iCPIs, such as anti-TIM-3, anti-OX40, anti-LAG-3, and anti-VISTA, respectively.

### Combination of Immunotherapies With Vaccines

Despite cellular therapies are still in their infancy compared to iCPIs the combination of different cellular therapies with antibodies directed against iCPs is promising and first results from *in vivo* models and clinical trials exist. Currently, a number of clinical trials using two or more combinations are investigated including different whole cell-based vaccines like tumor-infiltrating lymphocytes (TIL), T cell receptor (TCR), or chimeric antigen receptor (CAR)-modified T cells and dendritic cell (DC)-based vaccines (25). Interestingly, another novel approach is the co-delivery of PD-L1 siRNA with a DC-based mRNA vaccine, which caused a downregulation of PD-L1 in tumor-antigen presenting DCs thereby boosting anti-tumor responses (26). Despite preliminary investigations gave promising results, the major challenges of the combination of whole cell-based vaccines with iCPIs are adverse events due to toxicities and autoimmunity, which have to be reduced (27). It is also noteworthy that a synergistic effect of a synthetic DNA vaccine with antibodies directed against iCPIs was found, which was due to alterations of the immune regulatory environment (28).

### Combinations of iCPIs With IgG Antibodies

In addition to cellular therapies, the use of antibody dependent cell mediated cytotoxicity (ADCC) has recently been suggested as a promising combination with iCPIs (29). Immunoglobulin (Ig) G1 monoclonal antibodies (mAbs) have the highest capacity to induce ADCC in comparison to Ig isotypes (30–32). Thus, a number of IgG1 mAbs, such as Trastuzumab, Cetuximab and Rituximab, directed against the HER-2/neu, EGF-R, or the B cell-restricted antigen CD20, have been developed and were used for the treatment of different tumor types, such as colorectal cancer (CRC), head and neck squamous cell carcinoma (HNSCC), Non-Hogkin lymphoma and chronic lymphatic leukemia (CLL), respectively. These mAbs exert anti-tumor properties by inhibition of tumor growth, but modulation the immune cell activity (33–35). A combination of iCPIs with IgG1 mAbs can boost the innate and adaptive anti-tumor activity, recruit effectors, alters the composition of the TME by elimination of dysfunctional lymphocytes thereby enhancing the efficacy, durable responsiveness and patients' survival as shown for CRC and HNSCC (29). However, the inhibitor mediated ADCC and the recruitment of CD8^+^ cytotoxic T lymphocytes (CTL) to the tumor is associated with negative feedback loops, such as enhanced infiltration with Tregs and MDSC as well as an increased expression of different iCPIs (29). Thus, co-targeting of both immune suppressive mechanisms and the synergistic activity of e.g., Cetuximab and iCPIs might improve the outcome of patients. Indeed, a number of ongoing studies investigate the combination Cetuximab with various iCPIs including Avelumab in order to generate a beneficial immune effect.

## Combination of iCPI With Conventional Treatment and Increased Susceptibility of Tumor Cells to Lethal Signals From CTL Mediated by Death Receptors

### RT With Immunotherapy—and First Results

RT is used a standard treatment of many cancers by reducing the risk of recurrences after surgery as curative treatment of localized tumors or as palliative treatment to reduce the bulk of tumors. In addition, so called abscopal effects were demonstrated outside of the irradiated field (36). While RT can be immune suppressive, it can also enhance antigenicity and adjuvanticity by promotion of the release of tumor antigens (TA) combinations of immunotherapy with RT has been suggested (37–39). Although durable responses are rare, most patients benefit from this treatment by distinct mechanisms (40) including RT-mediated enhancement of T cell responses and changes in the TME composition. For example RT can reprogram the anti-myeloid TME to a pro-myeloid TME allowing recruitment of antigen presenting cells (APC) and T cells mediated by the induction of type I IFN due to activation of stimulator of interferon genes (STING) and its upstream signaling pathways. Cross presentation of tumor associated antigens (TAA) to CTL results in activation of T cells, which release IFN-γ known to increase and/or induce major histocompatibility complex (MHC) class I surface expression, (41–43) the factor associated with suicide (Fas) and the intracellular adhesion molecule-1 (ICAM-1) (44–46) involved in elimination of tumor cells. However, TFG-β is also released during RT, which inhibits immune responses by decreasing the capacity of DC to present TAA, T cell function, and HLA class I antigen expression on tumor cells thereby promoting tumorigenesis, which is associated with poor clinical outcome of patients (47). Other radiation induced cytokines, chemokines, and growth factors influence the balance between immune clearance and immune tolerance in the TME, play a dual role on the tumor infiltrating immune cell repertoire and on the modulation of anti-tumoral immune responses (48). In addition, RT can upregulate PD-1 and PD-L1 on tumor and immune cells (49, 50) providing a window of opportunity for anti-PD-L1/PD-1 inhibitor treatment by diminishing resistance to RT and upregulating the sensitivity to iCPI (51). Thus, iCPIs might work synergistically with the RT-induced T cell response through redundant pathways.

Indeed, preclinical data suggest that different immune modulators synergize with RT leading to tumor regression (52). For example, the combination of RT with an anti-CTLA-4 mAb not only increased the density of TILs and the CD8^+^ vs. CD4^+^ T cell ratio in a murine carcinoma model, but also resulted in a more oligoclonal T cell repertoire required to achieve tumor rejection thereby supporting the synergic activity of RT and CTLA-4 blockade (53). The feasibility combining focal radiation with systemic TGF-β blockade due to increased peripheral blood mononuclear cell (PBMNC) and central memory CD8^+^ T cell counts as well as abscopal effects was demonstrated in a metastatic breast cancer model (54). A combination of RT with STING agonists enhanced T cell priming and reduced tumor growth (41, 55). In sum, RT has the potential to efficiently induce the secretion of type I IFN and enhances the expression of the three prime repair exonuclease 1 (TREX1) known to degrade cytosolic double stranded DNA, which represents a key regulator of the cellular response to RT. TREX1 controls the immunogenicity of radiated cells by amplifying the immunogenicity of tumor cells and the abscopal responses (55).

Since tumors are often infiltrated by suppressive cells expressing high levels of PD-L1, high radiation doses might be more effective in eliminating tumor cells. So far, the dose and fractionation of RT in immune modulation has not been analyzed in detail. However, there exists evidence that the recruitment of DC to tumor cells is dependent on the RT dose and fractionation. Thus, RT must be administered at optimal doses, schedules and sequences to obtain a robust anti-tumor immunity.

In this context, a number of clinical trials have been completed, are currently recruiting or planned in different cancer types combining different FDA-approved iCPIs with radiotherapy including several radiation doses (56–58) (see ClinicalTrials.gov). Chemo-radiation combined with anti-PD-L1 antibody or placebo demonstrated a progression-free survival of PD-L1 antibody treated patients compared to placebo (59) demonstrating a beneficial effect of RT with iCPIs. This is further in line with improved anti-CTLA-4 mediated T cell responses upon *in situ* vaccination by RT (60). Furthermore, a combination of *in situ* vaccination with intra-tumoral injection of tumor-specific antibodies and systemic CTLA4 improved primary tumor response and survival in an experimental model (61).

### Combination of Chemotherapy With Immunotherapeutic Approaches

Chemotherapeutic agents (taxanes, cyclophosphamides) can promote anti-tumor immune responses by enhancing proinflammatory cytokines and exerting immune modulatory effects on tumor cells, such as e.g., an upregulation of HLA class I antigens, as well as on the TME, like depletion of myeloid-derived suppressor cells (MDSCs) and regulatory T cells (Tregs) (62). A major objective is to convert so called “cold” non-immunogenic tumors into “hot” immunogenic tumors, which are more sensitive to immunotherapy. Thus, chemotherapy-based immune modulation prior to iCPI treatment appears promising. In two experimental murine models of CRC (CT26) and RCC (RENCA) combining cyclophosphamide (CP) with CTLA-4 blockade had contrasting effects. In CT26-bearing hosts, CP augments the anti-tumor effect of ant-CTLA-4, while in RENCA this combination had only a marginal effect (63). In a clinical phase Ib study of advanced or metastatic NSCLC patients, Atezolizumab followed by chemotherapy significantly increased the response rate of NSCLC patients (NCT00527735) (64). Furthermore, 50 metastatic triple negative breast cancer (TNBC) patients treated with low-dose chemotherapy followed by Nivolumab demonstrated an increased overall survival (OS) with low toxicity, which was better than anti-PD-1 monotherapy (NCT02499367, J Clin. Oncol. 36, no. 15_suppl. (May 2018) 1012–1012; doi/10.1200/JCO.2018.36.15_suppl.1012). In sum, low-dosage immunogenic chemotherapies plus checkpoint blockers enhance tumor immunogenicity, which might revert tumor relapse by eliminating dormant cancer cells and thus should be used for combination therapies. This option was in line with results of a phase III clinical trial in NSCLC patients treated with Pembrolizumab in combination with chemotherapy, demonstrating a significantly increased OS and PFS compared to chemotherapy alone with an overall response rate of 48 vs. 19% with no change in adverse events (NCT02578680). Thus, this combination was synergistic and exhibits an acceptable safety profile (65, 66). It is noteworthy that OS of patients was improved independent of PD-L1 expression levels of tumor cells, which argue for combine iCPIs with chemotherapy replacing chemotherapy as the standard of care for first-line treatment of metastatic NSCLC.

## Combination of Immunotherapy With Targeted Therapies

Different growth factors and angiogenic factors and their receptors, including EGF/EGF-R, VEGF/VEGF-R, and angiopoietin, have been shown to affect the innate and adaptive immune response and induce immune suppression (12, 67). Based on the functions described, inhibition of these pathways might offer novel combination opportunities and synergies with iCPI (68). While the efficacy of immunotherapy is often associated with a reduced recruitment of blood vessels and activated cytotoxic T cells, anti-angiogenic therapy could reprogram the immune suppressive TME by normalizing the vasculature and increased T cell recruitment thereby enhancing the efficacy of cancer treatment. However, potent inhibition of angiogenesis increases hypoxia, which negatively interferes with immune responses (69). Thus, the anti-cancer immune response is impaired and vascular normalization by anti-angiogenic inhibitors could therefore revert the immune suppression and increase the efficacy of chemotherapy and immunotherapy (70–73). Indeed, a number of ongoing clinical trials combine anti-angiogenic therapy with iCPI alone or even in combination with chemotherapy (74). These combinations have synergistic effects and improve response rates, but some combinations exhibit a high toxicity (75).

Some targeted agents such as B-RAF and MEK inhibitors are associated with immune modulatory activity and might act synergistic with iCPIs. The BRAF inhibitor Vemurafenib was combined with anti-CTLA-4 mAb, but this combination had adverse effects, while combining Vemurafenib with a PD-L1 inhibitor and kinase/ERK inhibitor improved the treatment efficacy (76–78). Other therapeutic intervention with iCPIs include inhibitors directed against indoleamine (IDO) phosphoinositid-3-kinase-γ (PI3K-γ) (79), and colony stimulating factor 1, which lead in combination with anti-PD1 to a regression of all tumors and 90% survival after 90 days (80). Inhibitors directed against the molecules CDK4/6, which are essential for the initiation and development of breast cancer and ALL, demonstrated growth inhibition in various cancers as well as induction of anti-tumor immunity by enhancing TAA expression and inhibiting regulatory T cell proliferation (81, 82). Combining CDK4/6 inhibitors with iCPI treatment led to complete tumor regression and immunological memory in an experimental murine breast cancer model suggesting this treatment regimen as a promising option (83).

## Epigenetic Modulators

Epigenetic modulators, like inhibitors of histone deacetylases (HDAC^+^) or DNA methyltransferases (DNMT) are currently used for the therapy of different tumor entities. This is based on the fact that (i) HDAC is overexpressed in tumors, (ii) inhibition of HDAC negatively interferes with the expansion of MDSCs, (iii) tumor antigens (TA) are often methylated and thus not presented via HLA class I and class II molecules, and (iv) hypomethylating agents as well as HDACi have diverse immune modulating effects by e.g., upregulating HLA class I and components of the IFN-γ signal pathway (84). Since the different epigenetic modulators exhibit a low toxicity, the use of these inhibitors might increase the efficacy of single-agent immunotherapies (85). The combination of Ipilimumab with Nivolumab had synergistic effects on tumor growth with 5-azacytidine and entinostat and >90% of CRCs and 100% of metastatic mammary tumors were illuminated. Demethylation of the PD-1 promoter on T cells by 5-azacytidine in AML patients correlated with increased PD-1 expression, leading to the rational combination of DNMT inhibitor with anti-PD-1 mAbs, which are currently tested in clinical trials of NSCLC patients, but also of other cancers (86).

## Novel Strategies

Next to these options and based on the increased knowledge in the biology of cancer and immune cells and the composition of the TME, novel innovative strategies are currently discussed or studied in experimental models or clinical trials in conjunction with iCPIs. Some interesting strategies are depicted in the following part.

### Anti-KIR

Next to enhancing T cell mediated immune responses, natural killer (NK) cells are critical effectors of the innate immune system and are able to control tumor growth (87). This has been shown e.g., in acute myeloid leukemia (AML) patients, in which the number and activity correlated with relapse-free survival (RFS) (88). NK cells express both stimulatory, but also inhibitory receptors. The Ig-like KIRs prevent NK cell activation upon binding to their ligands, principally HLA-C molecules (89). The clinical relevance of KIR inhibition was demonstrated in models of allogeneic haplo-mismatched stem cell transplantation. In the absence of KIR/KIR-ligand binding, alloreactive NK cells were able to eliminate residual leukemia (90). Recently, a number of KIR antibodies have been developed that prevent the KIR/HLA-C interaction including e.g., IPH2101. This antibody augmented NK cell-mediated elimination of autologous human HLA-C^+^ AML blasts, which was confirmed in a NOD-SCID mouse model of NK cell-mediated tumor rejection. Since a correlation between PD-L1 and KIR expression was found in NSCLC and associated with a poor prognosis of these patients (91), a combination of anti-KIR and iCPIs is currently discussed.

### TLR Agonists

Toll-like receptors (TLR) agonists, such as SD101 and IV270 (92, 93) are under investigation in solid tumors (94, 95) due to their ability to induce potent anti-tumor immune response. TLR agonists could activate the innate immune response and revert immune suppression and tolerance (96, 97). Thus, it has been suggested to combine them with iCPIs to suppress tumor growth and shape the TME. First results in a murine HNSCC model demonstrated an increased ratio of M1/ M2 macrophages, T cell clonality and recruitment of CD8^+^ T cells (98).

### Modulators of Extracellular Matrix (ECM) in Combination With Immunotherapies

Components of the extracellular matrix (ECM) have been shown to play a key role in the initiation and progression of tumors by regulating different steps of the cancer process (99). Matrix components representing the matrisome in the TME are produced by mesenchymal stem cells (MSC), pericytes and cancer-associated fibroblasts (CAFs) and affect anti-tumor immune response and the efficacy of immunotherapies (100). ECMs have been shown to alter the TME and modulate the differentiation, migration, infiltration as well as polarization of immune cells in the TME (101). Thus, are involved in the development of an inflamed TME by regulating the activity of Tregs and immune suppressive myeloid cells (102). A combination of matrix metalloproteinase (MMP) inhibitors with an experimental mammary cancer model delayed tumor growth, reduced metastases formation and the percentage of Tregs and MDSCs as well as microvessel density (103). Thus, ECM components might serve as biomarkers to improve patients' stratification, but also could be used as therapeutic targets in combination with immunotherapies (104).

### Other Novel Potential Combination Partners for iCPIs

Based on the increased knowledge of the TME a number of potential novel co-targets for iCPI combinations have been suggested. These are the on the one hand approaches to normalize the host and immune cell metabolism by targeting e.g., cyclooxygenase (COX2). COX2 is overexpressed in different cancers (105, 106). The tumor-derived COX activity causes immune evasion. Interestingly, this could be reverted by a combination with iCPIs. Preclinical models demonstrated that COX inhibitors, such as aspirin, have a synergistic effect in combination with anti-PD1 in different murine experimental models leading to tumor eradication (107, 108).

Furthermore, exosomes known to transmit material from tumor cells to stroma and immune cells, which could lead to immune escape has been started to be therapeutically exploited either as nano-particle for drug delivery, direct exosome based immunotherapy, or removal of tumor-derived exosomes (TEX) from the periphery (109). On the other hand, it has been suggested to use TEX as tumor vaccines as a source of specific stimuli for anti-tumoral immune responses (110), but the clinical potential in monotherapy as well as in combination with other immunotherapies including iCPI has not yet been analyzed (111, 112).

## Biomarkers for Prediction of Response to iCPI and Combinatorial Therapies

Due to the limited durable response to iCPI and combinations, criteria to discriminate responders from non-responders prior to the initiation of treatment as well as during treatment are urgently needed. The predictive biomarkers might allow the selection of patients who are more likely to respond, while they might also allow to detect acquired resistance mechanisms. Some reports analyzed the composition of the intra-tumoral immune cell infiltration by IHC or multispectral imaging and the immune cell repertoire of peripheral blood lymphocytes for prediction of anti-tumoral immune responses (113–119). This was extended by high throughput RNA-seq, deep sequencing of TCR, mass cytometry as well as *in silico* analysis using TCGA data and a systematic bioinformatics pipeline leading to a multi-dimensional analysis of the immune signature regarding the responsiveness to iCPI therapy (120, 121). Based on these distinct approaches, an optimized immune marker panel, and interactive bioinformatics pipeline identified a responsiveness associated predictive signature in patients treated with anti-PD-1 immunotherapy (122). However, it has to be pointed out that there exists a high variability between traditional fluorescence flow cytometric analysis as well as mass cytometry. Therefore, a systematic prospective collection of peripheral blood from tumor samples is mandatory for determination of immune signatures in larger multi-center cohorts of patients treated with various iCPIs alone or in combination. This might lead to a prediction signature, which could be implemented in the clinical practice prior to immunotherapy.

## Conclusions

Blockade of iCPI has been successfully applied in a number of solid tumor and hematological neoplasms resulting in an enhancement of anti-tumor immune responses by targeting immune regulatory pathways. However, only a limited number of patients benefit from these therapies, which is often associated with toxicities and side effects of an autoimmune nature. Thus, prognostic and predictive biomarkers are urgently needed to define patients, which respond to given immunotherapy regimens with minimal toxicity. So far, tumor mutational burden (TMB), the immune cell infiltration as well as the expression of iCPs, such as PD-L1, have been discussed to be predictive for checkpoint blockade response. In addition, the list of combinations of iCPI with other therapies is extensive and despite the enthusiasm and potential of iCPI combination therapies, it further underlines the need to identify biomarkers in order to select patients undergoing immunotherapy combinations to provide a survival benefit for more patients and reduced adverse effects. Recent data suggest that next to the integration of TMB, iCPI expression and immune cell infiltration, host genetics, microsatellite instability, neo-antigen loss, the non-cellular composition of the TME, and the microbiome, should be analyzed. Thus, not only a standardized and optimized (immune)monitoring is crucial for tailoring immunotherapies, but it should also address the dynamics of immune response, posttranslational modifications, the contexture of immune and tumor cells as well as physical factors of the TME, e.g., hypoxia and pH. Overcoming these challenges and the implementation of new agents and combinatorial strategies are currently the major research focus in iCPI treatment to enhance their efficacy and avoid resistances.

## Author Contributions

The author confirms being the sole contributor of this work and has approved it for publication.

### Conflict of Interest Statement

The author declares that the research was conducted in the absence of any commercial or financial relationships that could be construed as a potential conflict of interest.

## Abbreviations

ADCC: antibody dependent cell mediated cytotoxicity
AML: acute myeloid leukemia
APC: antigen presenting cell
CAF: cancer-associated fibroblast
CDK4/6: cyclin-dependent kinase 4 and 6
CLL: chronic lymphatic leukemia
CP: cyclophosphamide
CRC: colorectal cancer
CTL: cytotoxic T lymphocyte
CTLA-4: T lymphocyte antigen 4
DC: dendritic cells
DNMT: DNA methyltransferase
ECM: extra cellular matrix
EGF: epidermal growth factor
EGF-R: EGF receptor
Fas: factor associated with suicide
FDA: Food and Drug Administration
HDAC: histone deacetylase
HDACi: HDAC inhibitor
HIF: hypoxia inducible factor
HNSCC: head and neck squamous cell cancer
ICAM-1: intracellular adhesion molecule-1
ICM: tumor extracellular matrix
iCP: immune checkpoint
iCPI: immune checkpoint inhibitor
IDO, indoleamine 2: 3 dioxygenase
IFN: interferon
Ig: immunoglobulin
IL: interleukin
KIRs: killer inhibitory receptor
LAG-3: lymphocyte-activation gene 3
mAb: monoclonal antibody
MDSC: myeloid-derived-suppressor cell
MHC: major histocompatibility complex
MMP: metalloproteinase
MSC: mesenchymal stem cell
NK: natural killer
NSCLC: non-small cell lung cancer
OS: overall survival
PBMNC: peripheral blood mononuclear cell
PD-1: programmed cell death protein 1
PD-L1: programmed cell death ligand 1
PFS: progression-free survival
RCC: renal cell carcinoma
RFS: relapse-free survival
RT: radiation therapy
sting: stimulator of interferon genes
TA: tumor antigen
TAA: tumor associated antigens
TAF: tumor associated fibroblasts
TAM: tumor-associated macrophages
TAN: tumor-associated neutrophil
TCR: T cell receptor
TEX: tumor-derived exosomes
TGF: transforming growth factor
TIL: tumor infiltrate lymphocyte
TIM-3: T cell immunoglobulin and mucin domain-3
TKI: tyrosine kinase receptor
TLR: toll-like receptor
TME: tumor microenvironment
TNBC: triple negative breast cancer
Treg: regulatory T cells
TREX1: three prime repair exonuclease 1
VEGF: vascular endothelial growth factor
VEGF-R: vascular endothelial growth factor receptor
VISTA: V-domain Ig suppressor of T cells activation.
